# Age-related thymic involution: Mechanistic insights and rejuvenating approaches to restore immune function

**DOI:** 10.1126/sciadv.aeb2970

**Published:** 2026-02-13

**Authors:** Jérémy C. Santamaria, Magali Irla

**Affiliations:** Centre d'Immunologie de Marseille-Luminy, CIML, CNRS, INSERM, Aix-Marseille Université, Marseille, Turing Centre for Living Systems, Marseille, France.

## Abstract

The gradual decline in thymic function with age, known as age-related thymic involution, leads to reduced T cell production, thereby increasing the risk of infections and cancer susceptibility and leading to poor vaccine responses. Moreover, T cell defects were recently involved in the age-related loss of tissue integrity and function. Mechanistically, thymic involution is driven by several factors, including hormonal modifications and chronic inflammation, leading to functional changes in the hematopoietic and stromal compartments. These progressive changes alter the cross-talk between developing T cells and thymic epithelial cells, which is pivotal for thymic function. Promising strategies to counteract thymic involution and rejuvenate immune T cell function have been recently identified. This review summarizes key insights into the underlying mechanisms of thymic involution and discusses current and emerging rejuvenation strategies to restore thymic function. These interventions show promise in regenerative medicine to promote healthy aging by alleviating age-associated immune decline.

## Teaser

Reversing age-related thymic decline restores immunity and paves the way for healthy aging.

## INTRODUCTION

**Fig. 1.**
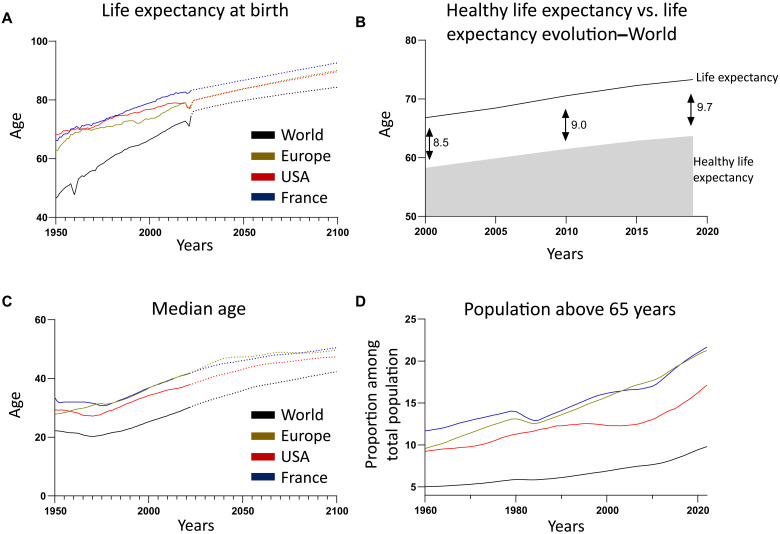
Worldwide demographic evolution. (**A**) Life expectancy at birth from 1950 to 2021 with projection from the United Nations to 2100. (**B**) Comparison of healthy life expectancy and global life expectancy at birth over time, worldwide. Arrows indicate the difference in years between these two conditions. (**C**) Median population age from 1950 to 2021 and projection from the United Nations to 2100. (**D**) Population above 65 years. Data are sourced from the World Data Bank and the WHO.

Global life expectancy increased by 6.3 years between 2000 and 2019 (Fig. 1A). However, healthy life expectancy at birth, defined as the period of life lived without chronic disease, increased by only 5.4 years over the same period. According to World Health Organization (WHO) data, this gap between life span (73.4 years) and healthy life span (63.7 years) has widened to 9.7 years representing a net loss of 1.2 years of healthy life between 2000 and 2019 (Fig. 1B). The aging of the population therefore represents a major public health challenge. Moreover, the global median age is projected to increase by 12 years by 2100 (Fig. 1C). In 2019, people over 65 years represented 10% of the world’s population, and this is projected to reach 22% by 2050, according to WHO estimates. This increase is even more pronounced in high-income countries, where already one-fifth of the population is over 65 (Fig. 1D). Since aging is the main risk factor for many human diseases, it is essential to promote healthy life expectancy, which would reduce medical costs (*1*–*3*).

Aging is a pathophysiological process characterized by a gradual loss of physiological integrity resulting from the accumulation of a wide variety of molecular and cellular damages. The increased incidence of diseases including cancer, diabetes, neurodegenerative and cardiovascular diseases, as well as cognitive and physical disabilities, is linked to chronic inflammation, termed “inflammaging” (*4*–*7*). Targeting the immune system holds substantial potential to mitigate aging hallmarks by breaking the cycle of chronic inflammation. Although inflammaging is primarily driven by the production of inflammatory molecules by nonimmune cells, it is also closely associated with the dysregulation of immune responses. It has recently been shown that induced premature senescence of immune cells alters immunity and promotes systemic aging, mimicking the natural process of aging (*8*). For instance, the transfer of artificially induced senescent immune cells or aged splenocytes into a young host led to senescence, while transfer of young splenocytes attenuated senescence. These findings support the idea that immunosenescence drives systemic tissue aging.

With age, the proportion of myeloid cells increases relative to lymphoid cells, which may be attributed to a differentiation bias in hematopoietic stem cells favoring the myeloid lineage (*9*–*12*). In addition, B cell production declines with age, which is associated with a reduction in naïve and mature B cells and an accumulation of anergic B cells (*13*–*15*). Moreover, age-related thymic involution contributes to a drastic decline in the production of T cells, further exacerbating this myeloid/lymphoid bias (*16*, *17*). All these defects contribute to impaired immune responses and increased susceptibility to various age-associated diseases. In addition to their protective roles against infections and cancer, T cells have been shown to recognize and eliminate senescent cells (*15*, *18*–*21*). Consequently, restoring lymphoid production, in particular of T cells, may be relevant for reducing inflammatory aging and delaying the onset of age-related diseases.

## HALLMARKS OF T CELL AGING

**Fig. 2.**
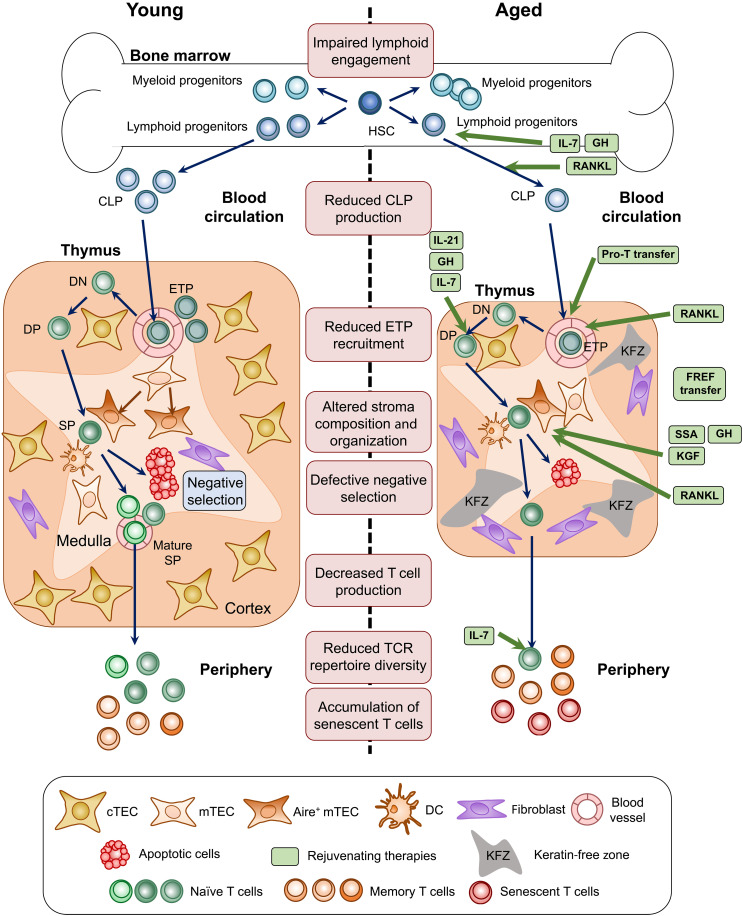
Alterations in T cell development during aging and molecules associated with thymic rejuvenation. Major alterations observed in the bone marrow and thymus, contributing to peripheral T cell defects with aging. Approaches to rejuvenate the thymic function are depicted. Pro–T cell transfer and interleukin-7 (IL-7) promote hematopoietic cell recovery, while FOXN1-reprogrammed embryonic fibroblast (FREF) transfer, keratinocyte growth factor (KGF), and sex steroid ablation (SSA) promote thymic epithelial cell (TEC) recovery. Growth hormone (GH) and receptor activator of nuclear factor κB ligand (RANKL) act on TECs, with RANKL also restores endothelial cell functional properties. CLP, common lymphoid progenitors; DC, dendritic cells; DN, CD4/CD8 double-negative cells; DP, CD4/CD8 double-positive cells; ETP, early T cell progenitors; HSC, hematopoietic stem cells; SP, CD4 or CD8 single-positive cells.

T cell responses rely on a highly diverse repertoire of T cell receptors (TCRs), enabling the recognition of a wide range of antigens. TCR diversity is established exclusively during T cell development in the thymus (*22*, *23*) (Fig. 2). However, this primary lymphoid organ undergoes severe alterations with aging, in both mice and humans, resulting in a progressive decline in T cell production and a reduced TCR diversity. This natural process, known as thymic involution, leads to a reduced production of recent thymic emigrants (RTEs) and thus to a rarefaction in naïve T cells (*17*, *24*–*26*). The decline in newly generated naïve T cells, along with the accumulation of antigen-experienced memory T cells, progressively narrows the TCR repertoire diversity in both naïve and memory T cell populations (*27*–*33*). Thymectomy at the young adult stage leads to an increased accumulation of senescence-associated T cells and accelerated homeostatic proliferation of peripheral T cells (*34*). Conversely, transplantation of an embryonic thymus into adult mice limits homeostatic T cell proliferation and delays the accumulation of senescence-associated T cells. These findings indicate that early loss of thymic function promotes premature T cell aging and that thymic involution plays a key role in the age-related accumulation of senescence-associated T cells. Beyond quantitative changes, RTEs and naïve T cells exhibit intrinsic defects that impair their function (*17*, *35*, *36*). Homeostatic proliferation contributes to the gradual accumulation of exhausted and senescent T cells showing altered functional properties that promote tissue senescence through the production of prosenescence factors, such as granzyme K (*18*, *29*, *37*–*39*). Senescent T cells develop a proinflammatory phenotype, called senescence-associated secretory phenotype, which contributes to chronic low-grade inflammation. Their proinflammatory cytokine profile promotes tissue damage and is linked to the progression of age-related diseases, including cardiovascular, neurodegenerative, and cancer diseases (*40*). While there is strong evidence of cell-intrinsic defects driving these quantitative and qualitative changes, T cell–extrinsic factors were also involved, as the aged microenvironment impairs the cellularity and function of young T cells following adoptive transfer or parabiosis (*18*, *36*, *41*–*43*). In contrast, the young microenvironment was unable to reverse the phenotype and function of aged T cells. Thus, both intrinsic and extrinsic factors alter T cell fitness in aged mice.

The reduced production of naïve T cells, coupled with alterations in the TCR repertoire, results in weakened responses to previously unencountered antigens. In addition to the decreased TCR signaling of naïve T cells, memory T cells show impaired survival, which hinders their ability to mount effective immune responses (*44*–*47*). Therefore, T cell–mediated immune responses become severely compromised during aging, leading to diminished protection against pathogens, tumors, and vaccines, while increasing susceptibility to autoimmunity (*48*). For example, only 30 to 40% of older adults are capable of mounting efficient immune responses upon influenza vaccination (*49*). In addition to dysfunctions in immune responses and consistent with the recognized role of T cells in immune surveillance of senescent cells, recent studies have emphasized the major role of T cells in maintaining physiological homeostasis and their contribution to age-related decline (*19*–*21*). For example, a recent study revealed an association between clonally expanded CD8^+^ T cells and senescence features in patients with Alzheimer’s disease (*50*). Furthermore, artificially induced T cell aging has been shown to promote tissue senescence and deterioration, leading to organ dysfunctions (*51*). These findings extend the role of T cells in age-related diseases, including cardiovascular, neurodegenerative, and metabolic disorders (*52*). Strategies to enhance immune response efficiency during aging are therefore appealing. However, targeting peripheral T cells may not be the most effective approach. While these treatments could potentially boost naïve T cells or enhance effector T cell functions, they are unable to restore alterations in the TCR repertoire. T cell aging was recently associated with thymic-related primary hallmarks, which are responsible for secondary hallmarks, including reduced T cell repertoire, naïve/memory imbalance, and T cell senescence. This cascade of events results in immune deficiency and inflammaging (*53*). Therefore, targeting the thymus to restore efficient T cell production with a broad TCR repertoire appears as a relevant therapeutic approach to renew and rejuvenate the peripheral T cell pool.

## AGE-RELATED THYMIC INVOLUTION

Thymic involution is characterized by a progressive reduction in thymic size, accompanied by a disruption of its architecture. This includes the loss of clear cortico-medullary demarcation, the emergence of epithelial-free regions, an increase in fibroblasts, and a gradual accumulation of adipocytes within the human thymus (*54*–*58*).

### Causes of thymic involution

While many physiological and environmental factors can affect the thymic size, its involution is a natural process that accelerates with age. Notably, the thymus exhibits a unique aging trajectory compared with other tissues, which is characterized by an early decline in function. Involution starts as early as 6 weeks in mice and during the first years of life in humans, reaching its peak at puberty (*16*, *59*–*61*). While thymic involution is a conserved process (*61*), a fundamental question remains: What is the biological reason for thymic involution? If it is detrimental in older adults, then why does it occur? One possible explanation is that negative effects in later life may be the trade-off for essential benefits in early life and adulthood. Thanks to medical advances, life expectancy in Europe and the United States has increased from 35 years in the 1800s to 80 years today. Therefore, previous generations likely died before exhibiting any sign of diseases related to thymic involution and immune senescence. Several theories have been proposed and are discussed in detail elsewhere (*57*, *62*). Given that thymic T cell development is energy intensive, limiting thymic function once peripheral T cell populations are sufficient may enable the redirection of energy to other biological processes. In addition, thymic involution may protect the organism by reducing the risk of self-reactive T cells escaping thymic tolerance, which can lead to autoimmune diseases. Because T cell development involves somatic rearrangements in TCR genes, this process is susceptible to favor the emergence of cancer cells. Thus, thymic involution may limit the onset of leukemia. Although this question is difficult to answer, one underestimated explanation is that thymic involution may be an epiphenomenon, resulting from other processes, with no notable advantages or disadvantages early in life. Since the negative effects of thymic involution manifest mainly after the reproductive period, this process may be conserved because it experiences minimal selection pressure. Thus, whether thymic involution has a biological function, and what that function might be, is still a matter of debate.

Numerous reports have highlighted the critical importance of maintaining the thymic activity, known as thymopoiesis, throughout life. First, age-related thymic involution, which leads to reduced T cell production, is tightly associated with an increased risk of infections and cancer (*48*). Second, young adults who underwent thymectomy in early childhood exhibit a decrease in CD4^+^ and CD8^+^ T cells, reduced proportions of naïve T cells, and an accumulation of oligoclonal memory T cells (*63*). Notably, their T cell compartment exhibits the characteristics typically observed in middle-aged and older individuals. Third, thymectomy in adults leads to a decreased number of signal joint TCR excision circles (sjTRECs), a reliable marker of T cell production, and to reduced TCR diversity. These patients have higher risks of death and cancer compared with control patients and the general population in the United States (*64*). They also had an increased risk of cancer relapse, while the risk of autoimmunity was only transient and modest. Overall, these findings show that impaired T cell production can result in serious health consequences and thereby highlight the critical importance of preserving the thymic function throughout life.

### Contribution of hematopoietic defects to thymic involution

In recent decades, substantial efforts have been made to uncover the mechanisms responsible for thymic involution, leading to the identification of several contributing factors. Prethymic events, particularly the reduction of circulating T cell progenitors, may be a major driver of thymic involution (Fig. 2) (*59*, *65*). While an increased number of hematopoietic stem cells has been observed in both aged mice and aged humans, these cells lose their capacity of self-renewal and exhibit reduced lymphoid potential, leading to a reduced production of common lymphoid progenitors (CLPs) (*9*–*12*, *66*). In bone marrow (BM) chimeras, aged BM precursors transplanted into young recipients generate CD4^+^ T cells with similar functional properties upon immunization to their young counterparts (*67*). Moreover, aged T cell progenitors retain a comparable ability to seed a fetal thymus, and aged thymi show a similar capacity to recruit these progenitors (*68*, *69*). However, early thymic progenitors (ETPs) and double-negative cells from aged mice exhibit impaired proliferation and elevated rates of apoptosis (*70*). A recent study indicates that the cellularity of ETPs, as well as CLPs in the BM and blood, declines as early as 3 months of age in mice (*59*), despite a conserved seeding capacity, as observed by others (*71*). Notch signaling is reduced in both the mouse BM and thymus from 3 months of age, which supports the idea that prethymic events and alterations in thymic stroma may initiate ETP reduction in adulthood. Furthermore, the aged thymus does not support the normal development of intrathymically injected young ETPs (*72*). These observations suggest that thymic involution is also mainly driven by changes in the thymic microenvironment, in addition to intrinsic defects in T cell progenitors. Given the continuous cross-talk between hematopoietic and stromal cells in the thymus (*73*–*76*), which controls the differentiation of both compartments, defining the exact cellular origin of thymic involution is challenging.

### Contribution of stromal defects to thymic involution

Among stromal cells, thymic epithelial cells (TECs) play a central role in T cell development by providing essential factors that control T cell proliferation, survival, and selection (*22*). In line with the notion that defects in TECs are a primary driver of thymic involution, manipulation of FoxN1 expression, the master regulatory factor for TEC differentiation, is sufficient to influence the thymic involution process. Reduced FoxN1 expression has been shown to accelerate thymic involution (*77*, *78*). Conversely, TEC-specific overexpression of FoxN1 delays thymic involution and the decline in naïve T cell production (*79*, *80*). In line with the reduced FoxN1 expression during aging, Wnt signaling, which regulates FoxN1 expression (*81*), has also been found to be reduced in the mouse and human thymus (*82*, *83*). Some reports suggest that altered Wnt signaling may contribute to the epithelial-mesenchymal transition (EMT), likely explaining the accumulation of fibroblasts, which subsequently differentiate into adipocytes in aged thymi (*84*, *85*). Notably, Wnt4 is down-regulated in TECs with age, while LAP2a is up-regulated, both events leading to peroxisome proliferator–activated receptor γ expression, controlling EMT and preadipocyte trans-differentiation (*85*). Therefore, impaired Wnt signaling may promote EMT, altering the support that TECs provide to developing T cells and accelerating thymic involution. In line with this notion, ablation of CD147 on T cells inhibits the EMT process in TECs, thereby preventing thymic involution (*86*). Similarly, increased insulin-like growth factor–binding protein 5 expression was associated with the EMT process, leading to reduced thymocyte proliferation and thus correlated with thymic involution in humans (*87*). Age-related deterioration of TECs is characterized by several alterations, such as reduced differentiation, survival, and proliferation. Notably, a decrease in the expression of cell cycle–associated genes, including the targets of the transcription factor E2F3, in TECs has emerged as a key hallmark of early thymic involution (*88*). Furthermore, the decline in TEC proliferation was associated with reduced expression of Myc target genes, and the induced Myc expression in adult TECs was sufficient to stimulate thymic growth and reverse involution (*89*, *90*). Two TEC subsets, called atypical age-associated TECs (aaTECs), namely, CLDN3^+^ aaTEC1 and PDPN^+^ aaTEC2, emerge with aging (*91*). These aaTECs, which form high-density clusters that are devoid of thymocytes, lack typical TEC markers and exhibit characteristics of EMT. These cells likely compete with TECs for growth or survival factors, acting as dysfunctional decoy cells. Therefore, aaTECs probably contribute to the disruption of thymic function during aging.

Reciprocally, differentiation and cellularity of TECs are controlled by developing T cells (*73*, *74*). By inducing TEC differentiation, the receptor activator of nuclear factor κB (RANK)–RANK ligand (RANKL) signaling axis controls the cross-talk between medullary TECs (mTECs) and thymocytes (*92*, *93*). Our team recently reported a decrease in RANKL expression during aging in several hematopoietic cell types, including γδ T cells, lymphoid tissue inducer cells, natural killer T cells, CD4^+^ single-positive (SP) thymocytes, and ETPs (*94*). In vivo neutralization of RANKL and inducible deletion of RANK specifically in endothelial cells have revealed that endothelial cells also rely on the RANK-RANKL axis for their cellularity and functional maturation. Therefore, the reduced availability of RANKL with age may contribute to thymic involution by altering the recruitment of circulating T cell progenitors via endothelial cells, which in turn alters the ability of TECs to support T cell development. In line with these findings, TEC cellularity strongly decreases with aging and is accompanied by an impaired differentiation, characterized by a progressive decline in mature mTECs expressing high levels of major histocompatibility complex–II, also called mTEC^hi^ (*54*, *95*). The expression of tissue-restricted antigens (TRAs) by mTECs is also reduced with age, thereby impairing the negative selection of potentially harmful self-reactive T cells (*95*).

Chronic inflammation also plays a key role in driving the thymic involution process. Increased levels of inflammatory markers have been observed in the thymus of both aged mice and patients (*96*). In particular, TECs and dendritic cells produce proinflammatory cytokines, such as interleukin-1 (IL-1), IL-6, macrophage colony-stimulating factor, and tumor necrosis factor–α, suggesting that the increasingly inflammatory microenvironment contributes to thymic atrophy. Accordingly, the administration of IL-1α, IL-1β, or IL-6 in mice accelerates thymic involution in a dose-dependent manner, highlighting their detrimental effects on thymic structure and function (*96*, *97*). The proinflammatory signature of the thymic microenvironment may be triggered by the inflammasome, involving NLRP3 (nucleotide-binding oligomerization domain–like receptor family pyrin domain containing 3) that recruits the adaptor protein Asc (apoptosis-associated speck-like protein containing a caspase activation and recruitment domain) and the enzyme caspase-1, which then cleaves pro–IL-1β into its active form, IL-1β. Mice deficient in *Nlrp3* or *Asc* exhibit reduced thymic involution, characterized by an increase in cortical TECs (cTECs), T cell progenitors, and TCR repertoire diversity (*98*).

Reactive oxygen species, contributing to damage in many tissues, seem to be implicated in thymic involution. In particular, TECs are sensitive to oxidative DNA damage (*99*). Genetic complementation of the hydrogen peroxide–reducing enzyme catalase in stromal cells reduces thymic atrophy but does not fully prevent it. However, despite delaying thymic atrophy and mitigating the declining production of influenza-specific T cells, catalase overexpression does not restore the decrease in TRA expression and defective negative selection (*100*). In addition, the administration of the mitochondria-targeted antioxidant SkQ1 (plastoquinonyl decyltriphenyl phosphonium) seems to inhibit thymic involution in Wistar normal and senescence-prone rats (*101*).

Deficiencies in nutrients, including vitamins and minerals, such as zinc, iron, and magnesium, can also contribute to thymus atrophy (*102*–*105*). The age-dependent reduction in plasma zinc levels is linked to thymic involution, due to reduced thymulin activity (*106*). Oral zinc supplementation in aged mice regenerates the thymus, which was characterized by the restoration of the TEC network (*104*, *105*). While oral supplementation with high-dose zinc regenerates the thymic function and the production of naïve CD4^+^ T cells in patients undergoing hematopoietic stem cell transplantation (*107*), its benefits for thymic regeneration in older adults remain to be demonstrated. Similarly, a deficiency in vitamin D can accelerate the thymic involution process. Mice deficient for *Cyp27b1*, which cannot produce the hormonally active form of vitamin D (1,25-dihydroxyvitamin D), exhibit premature thymic aging, characterized by skewed mTEC differentiation, reduced Aire and TRA expression, and consequently impaired negative selection (*108*).

MicroRNAs (miRNAs) have also recently been implicated in thymic involution (*16*). In particular, the expression of miR-181a-5p, which interferes with transforming growth factor–β (TGFβ) expression and signaling, decreases in TECs from aged mice. This reduced expression promotes TGFβ-mediated inhibition of TEC proliferation (*109*, *110*). In contrast, miR-125a-5p expression increases in TECs during aging and down-regulates FoxN1 expression, thereby accelerating thymic involution (*111*). Furthermore, more than a hundred miRNAs were significantly up- or down-regulated between newborns and 70-year-old men, many of which have been identified as modulators of the Wnt pathway (*83*).

In addition to age-related involution, many factors can influence thymic size throughout life such as infections, stress, hormonal variations, pregnancy, and cytoreductive therapies (*16*, *97*). In humans, beyond genetic factors, individual life experience and environmental cues contribute to the variable rate of age-related thymic involution. Therefore, decoding the mechanisms of thymic involution is essential for developing personalized therapeutic strategies aimed at restoring T cell production in aged individuals.

## STRATEGIES FOR THYMIC REJUVENATION

From a therapeutic perspective, numerous molecules have been tested to restore thymic function, mainly following conditioning treatments in the context of hematopoietic stem cell transplantation (*55*, *112*–*114*). Some of them have also been used to rejuvenate the thymus during aging, by targeting either developing T cells or stromal cells, particularly TECs (Fig. 2). Alternatively, cell- and tissue-based therapies represent promising strategies for restoring thymic function.

### Molecules acting on developing T cells

The expression of IL-7, a γ-chain cytokine, expressed by TECs that is crucial for thymocyte survival and proliferation, declines in the thymus with age (*115*, *116*). IL-7 administration increased the viability and numbers of CD3/CD4/CD8 triple-negative thymocytes in 22-month-old mice (*117*). An IL-7–CCR9 fusion protein, designed to enhance thymus targeting following intravenous administration, modestly increased the number of developing T cells but significantly improved CD8^+^ T cell responses to influenza infection in 20-month-old mice (*118*). However, in a trial on rhesus macaques, the response to recombinant IL-7 was transient, and no long-term benefits were observed in the naïve T cell population of aged macaques (*119*). Recombinant IL-7 was found to enhance the short-term expansion of naïve and memory T cells independently of the thymic function in mice (*120*). Accordingly, clinical trials involving hematopoietic stem cell transplantation have shown that IL-7 primarily stimulates peripheral CD4^+^ and CD8^+^ T cells (*121*), with no apparent effects on thymic function, since the levels of RTEs and TRECs measured in blood were not increased in aged patients (NCT00684008) (*122*).

Administration of IL-21, another γ-chain cytokine produced by TECs, to 15-month-old mice has been shown to increase T cell development and the production of circulating RTEs. IL-21 treatment in aged mice enhanced the response to vaccination with melanoma antigen–pulsed dendritic cells, thereby delaying the growth of B16 tumors without inducing signs of autoimmunity (*123*).

Thymosin α1 is a peptide, derived from prothymosin α, which is naturally produced by TECs and has the ability to increase the number and function of T cells (*124*). Thymosin α1 is used to treat some chronic viral infections, such as hepatitis B and C, as an immunomodulator (*125*). In a recent retrospective analysis of patients with COVID-19 treated with thymosin α, this hormone enhanced T cell production, as measured by increased circulating TRECs and elevated numbers of peripheral CD4^+^ and CD8^+^ T cells, indicating improved thymic function (*126*, *127*). This treatment resulted in a 60% reduction in mortality among patients with severe COVID-19.

### Molecules acting on TECs

Since alterations in the stromal microenvironment contribute to thymic involution, restoring its cellular composition and function has emerged as a promising strategy for rejuvenating the thymic function.

The age-related decline in the expression of the transcription factor FoxN1 contributes to thymic involution (*79*, *80*). Consistently, intravenous injection of recombinant FoxN1 protein fused with an N-terminal CCR9 domain to enhance thymus targeting has been recently shown to increase TEC cellularity and improve thymopoiesis in 14-month-old mice (*128*). These findings suggest that the administration of recombinant proteins targeting the thymus could be a promising strategy to promote thymus rejuvenation. Alternatively, intrathymic injection performed under endoscopic guidance or the intrathymic delivery of drug vectors by electroporation may constitute other alternatives (*129*–*131*).

Exogenous administration of keratinocyte growth factor (KGF), also known as fibroblast growth factor 7 (FGF7), in 15- and 18-month-old mice has been shown to efficiently restore TEC cellularity and organization (*132*). KGF acts directly on TECs, which express its FgfR2IIIb cognate receptor, stimulating their proliferation and thereby their regeneration (*133*). This resulted in increased T cell development and T cell–mediated antibody production, with an effect lasting for up to 2 months after a single injection cycle (*132*). However, in a clinical trial (NCT01712945) aimed at evaluating the potential of recombinant KGF (palifermin) to restore T cells in patients with relapsing-remitting multiple sclerosis, administration of KGF to 18- to 50-year-old individuals unexpectedly resulted in a reduction of circulating naïve T cells, RTEs, and sjTREC levels, leading to trial termination (*134*).

Sex hormones, particularly androgens and estrogens, play a key role in thymic involution. Increased levels of androgens and estrogens during puberty are strongly associated with the onset of thymic involution. Androgen deficiency in male mice has been shown to ameliorate thymic development by acting on TECs (*135*). Castration significantly restores the cellularity of ETPs, enhances T cell development, and boosts the production of RTEs as well as T cell responses in both 9-month-old middle-aged mice and 2-year-old mice (*136*, *137*). Individuals over the age of 60 undergoing sex steroid inhibition, as part of prostate cancer therapy, exhibited increased total and naïve circulating T cell numbers, along with elevated TREC levels (*136*). However, another study revealed that although castration induced efficient but transient thymic regeneration, the expression of TRAs in TECs was not restored, raising concerns about the potential escape of autoreactive T cells from the thymus (*82*). This observation is consistent with the fact that, unlike estrogen, which inhibits Aire expression in both mice and humans, testosterone promotes Aire expression, thereby supporting negative selection of autoreactive T cells (*138*). Therefore, although effective, sex hormone ablation should be used with caution because of the potential risk of autoimmunity arising from imperfect TEC regeneration and impaired negative selection of autoreactive T cells.

The decline in growth hormone (GH) levels with age has also been associated with thymic involution (*139*). Although GH receptors are expressed on both TECs and thymocytes, GH effects are primarily mediated through the expression of insulin-like growth factor 1 (IGF-1), which is predominantly produced by TECs (*140*, *141*). While both thymocytes and TECs express the IGF-1 receptor, IGF-1–mediated enhanced thymic function is primarily attributed to the expansion of TECs (*142*). A recent exploratory study in healthy 51- to 65-year-old men found that treatment with recombinant GH, in combination with metformin and dehydroepiandrosterone, leads to a reversal of thymic fat accumulation (*143*). In line with potential thymic rejuvenation, naïve T cell numbers were increased, and epigenetic age estimation, measured in peripheral blood mononuclear cells, was reduced. However, a substantial portion of the measured parameters returned to baseline 6 months after discontinuing treatment. A follow-up clinical trial is currently recruiting volunteers to extend the study (NCT04375657), which may represent a major step in thymic rejuvenation therapies aimed at restoring naïve T cells in humans.

Moreover, numerous stress stimuli that trigger adrenal production of glucocorticoids, such as psychological stress, fasting, intoxication, and infection, can rapidly induce thymic involution (*144*). All thymocytes express the glucocorticoid receptor at varying levels, with DP thymocytes being particularly sensitive to glucocorticoid-induced apoptosis (*145*). Nevertheless, because DP thymocytes are continuously replenished, the thymus can recover rapidly once glucocorticoid levels return to basal levels.

Caloric restriction in mice prevents increased thymic adipogenesis and leads to the maintenance of cortical and medullary cell density and the preservation of epithelial signatures (*146*). The reduction in EMT and thymic adipogenesis led to increased T cell production and prevented TCR repertoire restriction. A recent study has shown that middle-aged adults undergoing a 2-year caloric restriction intervention showed thymic rejuvenation, characterized by enhanced production of naïve T cells and improved immune profiles (*147*). Youm and colleagues (*148*) reported that caloric restriction promotes the expression of the peptide hormone FGF21, which declines with age. This study also showed that FGF21 overexpression prevents age-related thymic involution, whereas FGF21 deficiency accelerates thymic atrophy. Recent studies further demonstrated that FGF21 overexpression in TEC or adipocytes delays thymic involution and improves T cell development and selection (*149*, *150*). However, it remains unclear whether FGF21 can reverse thymic involution once established in aged animals. While human studies remain limited, these findings are encouraging and suggest a translational potential for caloric restriction as a strategy to enhance immune function in aged adults.

The RANK-RANKL signaling axis is implicated in TEC differentiation at a steady state as well as in TEC regeneration upon BM transplantation after lethal total body irradiation (*92*, *151*). In the context of aging, the gradual decrease in RANKL expression in the thymus contributes to the involution process (*94*). Although the reasons for this decrease in RANKL expression require further investigation, a plausible explanation is that altered TGFβ receptor II (TGFβRII) or TCR signaling may contribute, since TGFβRII stimulation in synergy with TCR activation can up-regulate RANKL expression in CD4^+^ SP thymocytes (*152*, *153*). The intravenous administration of RANKL protein in aged mice rejuvenates the thymic function by ameliorating (i) thymic architecture, (ii) the cellularity of TECs and thymic endothelial cells, (iii) T cell progenitor mobilization from the BM and homing to the thymus, and thereby (iv) T cell production (*94*). The use of RANKL is particularly interesting, as it stimulates both TECs and endothelial cells that express its cognate receptor, RANK. Therefore, RANKL treatment enhances TEC niches and promotes the recruitment of T cell progenitors via endothelial cells, both events contributing to efficient thymic rejuvenation. Given the role of the RANK-RANKL axis in osteoclast differentiation (*154*), the use of recombinant RANKL protein should be carefully adjusted in terms of dosage and frequency of administration to prevent the risk of inducing osteoporosis in older individuals. RANKL-mediated thymic rejuvenation enhances immune responses to immunization and improves antimelanoma T cell responses that are characterized by increased infiltration of effector cytotoxic T lymphocytes (*94*). RANKL also stimulates both endothelial cells and TECs in human thymic organocultures (*94*). Thus, RANKL offers a promising strategy in regenerative medicine to restore T cell immunity.

### Cell therapy–based strategies

Given that the decline in circulating T cell progenitors and thymic ETPs in aged mice may contribute to thymic involution (*59*, *70*), the transfer of in vitro–generated T cell progenitors could represent a promising strategy to rejuvenate thymic function (*155*). Moreover, given that T cell progenitors express RANKL, they may also contribute to stroma rejuvenation (*94*, *156*).

Adoptive transfer of regulatory T cells from young mice (6 weeks) into aged mice (24 months) enhances thymic regeneration following sublethal total body irradiation, through their secretion of amphiregulin, an epidermal growth factor receptor ligand (*157*). The potential of amphiregulin or regulatory T cell–based interventions to rejuvenate thymic function in aged mice may represent a valuable strategy that warrants thorough evaluation.

Since thymic involution is closely associated with the decline in TEC cellularity and function, strategies aimed at supplementing the aged thymus with functional TECs are expected to restore T cell production. The intrathymic engraftment of fetal or neonatal TECs into middle-aged mice (9 to 12 months old) promotes thymic growth and increases T cell production (*158*). Another study also demonstrated that the intrathymic injection of in vitro–generated FoxN1-reprogrammed embryonic fibroblasts in 20-month-old mice induced thymic growth and improved its architecture, thereby increasing T cell production (*159*). This approach partially restored negative selection by enhancing Aire expression in mTECs. It also attenuated peripheral inflammaging and decreased lymphocyte tissue infiltration.

Another promising approach is the transplantation of cultured human thymic epithelium obtained from thymic tissue excised during heart surgery in neonates and infants. The feasibility of using this approach has been demonstrated in patients with athymic complete DiGeorge syndrome, characterized by a profound T cell deficiency (*160*). Alternatively, grafting human thymic organoids, reaggregated from human primary ETPs and TECs derived from induced pluripotent stem cells of the recipient, is an attractive strategy for a clinical-grade approach (*161*–*163*). This opens exciting and promising opportunities for rejuvenating thymic function.

## CONCLUDING REMARKS

**Fig. 3.**
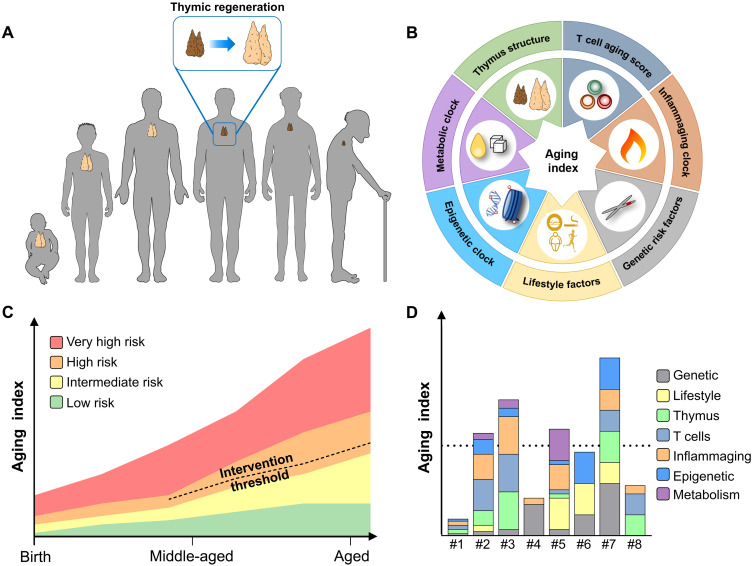
Combination of biomarkers and aging metrics to identify patients at risk who may require rejuvenation interventions. (**A**) Thymic rejuvenation is an attractive approach to prevent age-related diseases and should therefore be used as a prophylactic treatment for middle-aged individuals. (**B**) The combination of several biomarkers and metrics should help to build an aging index to predict patients with high risks to develop age-associated diseases. (**C**) This index would allow to define risks and an intervention threshold according to patient’s chronological age. (**D**) This strategy would allow to stratify patients (as exemplified here for eight individuals) of an identical chronological age and identify those eligible to rejuvenating therapies. Dashed lines indicate the intervention threshold.

The gradual decline in T cell production with age substantially contributes to impaired anti-infectious and antitumor responses, along with an overall deterioration of tissue integrity. This underscores the importance of developing therapeutic approaches aimed at reversing the process of thymic aging to prevent age-related pathologies due to abnormal T cell responses (Fig. 3A). Although numerous factors influencing thymic involution have been identified, the overall intrication of the underlying mechanisms remains poorly understood. Given that alterations in TECs and their complex interplay with developing T cells play a crucial role in thymic involution, further investigations are needed to identify effective therapeutic targets to rejuvenate T cell production. A deeper understanding of TEC alterations is particularly important in light of the recent discovery of their high heterogeneity (*164*).

Moreover, further studies are needed to determine whether the duration of thymic rejuvenation is important and whether combined therapies targeting both developing T cells and TECs provide additional benefits for thymic rejuvenation. In addition, combining strategies to restore thymic function with conditioning regimens aimed at increasing niche space and promote the expansion of newly developed T cells could enhance the efficiency of peripheral T cell renewal. Alternatively, combining strategies to restore thymic function with interventions targeting senescent cells that contribute to tissue damage using small-molecule senolytic drugs, currently being tested in clinical trials, could represent a groundbreaking approach for treating age-related pathologies in the future (*165*). This approach will have several beneficial effects by providing space for newly developing T cells capable of restoring efficient immune surveillance and reducing the secretion of inflammatory mediators by senescent cells, thereby limiting chronic inflammation.

Such advances are expected to pave the way toward developing prophylactic strategies to prevent age-related immune defects in humans. The thymus is sensitive to various factors, including chronic stress, hormonal variations, infections, and exposure to radiation or chemotherapy, leading to different rates of involution among individuals (*16*, *97*). Echographic analyses of the thymic three-dimensional structure, using, for example, computed tomography scans, should help in assessing the extent of thymic involution and facilitate the identification of patients who may benefit from a thymic rejuvenation protocol. Since an individual’s genetic background and life history influence immune system aging (*166*–*169*), the use of biomarkers is essential for identifying those at higher risk of age-related diseases and may benefit from rejuvenating interventions. In this context, the immune aging (IMM-AGE) score, which assesses the dynamics of immune cell populations with age (*170*), and the inflammatory aging clock (iAGE), based on the levels of circulating immune proteins, have been developed (*171*). Both metrics predict morbidity and mortality, which is promising for clinical use. Moreover, evaluating genetic variations would also serve as an important indicator of the risk for age-related diseases. For instance, allelic variations in genes encoding for *FOXO3* (Forkhead box O3) and *APOE* (apolipoprotein E) were identified to be tightly linked to human longevity and healthy aging (*172*, *173*). Numerous other genes are suspected to be linked to longevity or frailty but are still under investigation (*166*, *169*). Epigenetic clocks, which mainly assess DNA methylation patterns that are heritable traits, represent important metrics for evaluating the biological age of individuals using blood and tissue samples (*174*, *175*). Given that T cell aging is influenced by numerous factors that vary substantially between individuals, the development of a large-scale aging index is crucial to identify patients at higher risk (Fig. 3, B to D) (*176*). This would ultimately enable a personalized medicine approach to promote healthy aging.
